# Dietary Supplementation of Yerba Mate (*Ilex paraguariensis*) during the Dry Period Improves Redox Balance in Lactating Dairy Cows

**DOI:** 10.3390/antiox8020038

**Published:** 2019-02-09

**Authors:** Olimpia Barbato, Belinda Holmes, Iulia-Elena Filipescu, Pietro Celi

**Affiliations:** 1 Dipartimento di Medicina Veterinaria, Università degli Studi di Perugia, Perugia 06123, Italy; olimpia.barbato@unipg.it (O.B.); filipescu.iuliaelena@yahoo.com (I.-E.F.); 2 Faculty of Veterinary Science, University of Sydney, Narellan, NSW 2567, Australia; bhol5217@uni.sydney.edu.au; 3 Animal Nutrition and Health, DSM Nutritional Products, Kaiseragust 4303, Switzerland

**Keywords:** Yerba Mate, redox balance, dairy cattle, antioxidants

## Abstract

Thirty-six pregnant Holstein–Friesian cows were used to study the effect of Yerba Mate (YM) supplementation during the dry period on redox balance. The treatments groups were Control (no YM), YM 250 (250 g/cow/day), and YM 500 (500 g/cow/day). Blood samples were obtained 30 days prepartum, at calving, and monthly postpartum until four months post calving. Liveweight (LW) and body condition score (BCS) were assessed prepartum, at calving, and then postpartum monthly until the end of the trial. Plasma was analyzed for hydroperoxides (d-ROMs), advanced oxidation protein products (AOPP), and biological antioxidant potential (BAP). The oxidative stress index (OSI) was calculated as OSI = ROMs/BAP × 100. Cows were milked twice daily, and milk yield data were recorded daily. Redox balance was improved by YM supplementation, as reflected in the lower OSI values observed in the YM groups. Yerba Mate supplementation significantly affected LW, but did not affect BCS. Milk yield averaged 28.1 ± 0.40, 29.0 ± 0.48, and 29.9 ± 0.46 L/cow/day in the Control, YM 250, and YM 500 groups, respectively, but was not significant. Nutritional manipulation during the dry period with Yerba Mate has demonstrated the potential to improve redox balance and milk yield.

## 1. Introduction

The transition period of dairy cows generally refers to the period of three weeks pre-prepartum to three weeks post-partum, and is particularly important for dairy cow health and subsequent performance [1]. The transition period is also characterized by a depleted antioxidant status, often resulting in oxidative stress [2,3]. Dietary antioxidants are crucial to maintain good production performances and optimal immunity and health in ruminants [4]. Oxidative stress, resulting from increased production of pro-oxidants, and/or a decrease in antioxidant defense, leads to the damage of biological macromolecules and disruption of normal metabolism and physiology [5]. Oxidative stress has been associated with diseases [6] and may contribute toward reproductive problems [7] and metabolic diseases [5] in dairy cows. Several trace elements and vitamins are potentially useful in maintaining an appropriate balance of antioxidants in the dairy cow to cope with the increased production of pro-oxidants around parturition. 

Supplementation of dairy cows with exogenous dietary antioxidants has the potential to ameliorate the negative consequences of oxidative stress by scavenging peroxides [4]. The potential to increase milk production and improve health and reproduction has made nutritional manipulation of the periparturient cow a focus for research [8]. Correct nutrition of the transition cow is critical to ensure maximum dry matter intake, good health, increased reproductive efficiency, and optimum milk production in the subsequent lactation [9]. Nutritional manipulation with alternative feedstuffs during the pre-partum phase of the transition period represents an ideal strategy to achieve these objectives. 

Yerba Mate (*Ilex paraguariensis*) is known to possess a strong antioxidant capacity due to its high concentration of polyphenols such as chlorogenic acid [10] and caffeoyl derivatives such as caffeic acid [11]. Yerba Mate is a very potent inhibitor of oxidative stress [12]. Additionally, compounds such as condensed tannins have been identified in Yerba Mate [13]. Studies on the effect of Yerba Mate supplementation in ruminants have shown that Yerba Mate supplementation increased feed intake and wool growth in lambs [14], increased milk solids in ewes [15], and sustained milk yield in mid-lactating cows [16], while its administration in growing calves can influence lipid metabolism [17]. Recently, it has been observed that Yerba Mate is an antioxidant source for increasing reducing power in milk from dairy cows [18]. Therefore, the objective of this study was to investigate the effect of Yerba Mate supplementation during the pre-partum phase of the transition period on redox balance in lactating dairy cows. The hypothesis of this study was that increasing levels of Yerba Mate in the diet of dairy cows during the dry period will improve their redox balance following parturition.

## 2. Materials and Methods 

### 2.1. Animals, Location, Husbandry, and Experimental Protocol

The study was conducted at the Corstophine Dairy, Faculty of Veterinary Science, Camden Campus, from February (Month −1) through to September (Month 6) 2010. Approval from the Animal Ethics Committee of the University of Sydney (N00/5-2008/1/4812) was obtained before commencing this study. A total of 36 pregnant Holstein–Friesian cows of mixed parity (1–7), homogenous for age (age; 6.5 ± 1.65 years), body condition score (BCS; 3.3 ± 0.2), and liveweight (LW; 680 ± 60kg), were enrolled in the study and assigned to three dietary treatments in a randomized control study. The treatments were control (no additional supplement, *n* = 9), control diet plus 250 g/cow/day Yerba Mate (*n* = 16), and control diet plus 500 g/cow/day Yerba Mate (*n* = 17). Dose rates were extrapolated from previous studies in lactating dairy cows [16]. The dried leaves of Yerba Mate were pelleted and administered once a day when cows were brought to the dairy to receive their pre-partum allocation of concentrate. Cows were fed in individual feed troughs to prevent feed contamination. The nutritional composition of the Yerba Mate was as follows: 94% dry matter (DM), 16% crude protein (CP), 21% neutral detergent fibre (NDF), 13% acid detergent fibre (ADF), 1.9% water soluble carbohydrates (WSC), and 11.73 MJ/kg DM of metabolizable energy (ME) and 0.1% condensed tannins. Yerba Mate also contained 9.8 mg/100 g of caffeine. Cows received the dietary treatments once daily for approximately 30 days before the expected date of calving. After calving, cows were managed as a single herd and consumed a typical pasture-based diet, which consisted of kikuyu grass (*Pennisetum clandestinum*), oversown with short rotation ryegrass (*Lolium multiflorum*), and perennial ryegrass (*L. perrene*)/white clover (*Trifolium repens*). The cows had access to pasture between the two milkings and were grazed in accordance with the best practice of using pasture on offer and leaf stage as the criterion to flag time to graze.

Prior to parturition, dry cows received their concentrate (4 kg/cow/day) of Elite Plus, Weston Animal Nutrition. Following parturition, the concentrate allowance was 8 kg/cow/day in early lactation, 6 kg/cow/day in mid lactation, and 5 kg/cow/day in late lactation, allocated twice daily at milking. The composition of the Elite Plus pellets was as follows: 84.6% DM, 18% crude protein, 22.7% neutral detergent fibre (NDF), 8.7% acid detergent fibre (ADF), 6.4% water soluble carbohydrates, and 13 MJ/kg DM of metabolizable energy (ME). The pellets also contained 0.0044 MIU/kg of vitamin A, 0.001 MIU/kg of vitamin D_3_, 0.007 g/kg of vitamin E, 2% calcium, 0.5% phosphorous, 0.7% sodium, 0.7% chloride, 0.7% magnesium, and 0.1% sulphur. Because of the limited pasture availability during this time of the year (March to July), maize silage (*Zea mays*) was supplemented to the cows (5 kg DM/cow/day) after the afternoon milking.

Following parturition, cows were moved into the lactating herd and milked twice daily at 05:00 and 14:00. Cows were milked using a DeLaval automated milking machine, where individual milk yield was automatically measured on a daily basis and recorded using the computer-based software DeLaval Alpro dairy management system. Milk yield data were subsequently pooled weekly for the purpose of statistical analysis; milk yield was monitored for 30 consecutive weeks. Cows were weighed prior to parturition (Month −1), and each month following calving until September (Month 6). On these occasions, the body condition score of the cows was scored using the five-point body condition score (BCS) scale (1 = thin; 5 = fat) [19].

### 2.2. Blood Sampling and Assays

Blood samples were obtained from each cow via the middle caudal vein in BD Vacutainer® collection tubes containing Li-heparin as anticoagulant a month prior to parturition (Month −1), at calving (Month 0), and then post-partum in April (Month 1), June (Month 3), and July (Month 4). Blood samples were shielded from light and then maintained on ice during collection and transportation to the laboratory. Plasma was isolated from blood by centrifugation immediately after sampling at 1500× *g* for 10 min at 4 °C. The supernatant plasma was stored in a freezer for later analysis of reactive oxygen metabolites (ROMs), advanced oxidation protein product (AOPP), and biological antioxidant potential (BAP). 

Free oxygen radicals were measured using the concentration of ROMs as determined by a colorimetric assay on plasma (d-ROMs Test, Diacron International, Grosseto, Italy). This test measures the concentration of hydroperoxides such as hydrogen peroxide, generated by the oxidation of glucosides, lipids, amino acids, peptides, proteins, and nucleotides [20]. In the presence of free iron, hydroperoxides can generate free radicals, and are thus considered specific markers of oxidative damage. In the d-ROMs test, reactive oxygen metabolites, in the presence of iron released from plasma proteins by an acidic buffer, generate alkoxyl and peroxyl radicals, which in turn oxidize an alkyl-7 substituted aromatic amine (*N,N*-dietylparaphenylendiamine), producing a pink-colored derivative that can be photometrically quantified at 505 nm [21]. The results are expressed in ‘Carratelli units’ (UCarr), where 1 UCarr corresponds to 0.08 mg/100 mL of hydrogen peroxide.

Plasma concentration of AOPP was estimated according to Witko-Sarsat et al. [22]. AOPP were measured by spectrophotometer (FLUOstar Optima, BMG LABTECH HmbH, Mornington, Australia) at 340 nm. Two hundred microlitres of plasma diluted 1/5 in PBS (5 mM; pH7.2) was placed into wells of a 96-well plate followed by the addition of 20 µL of glacial acetic acid (Fluka 45732). A standard curve ranging from 0 to 200 µmol/litre was made using a chloramine-T solution (Sigma-Aldrich). To the standard wells, 10 µL of 1.16 M of potassium iodide (Sigma-Aldrich ReagentPlus^®^, Castle Hill, Australia) was added to 200 µl of the chloramine-T solution, followed by 20 µL of acetic acid. Immediately after (within 5 minutes), the absorbance of the reaction mixture was read. AOPP concentrations were expressed as micromoles per litre of chloramine-T equivalent.

Plasma antioxidants were quantified by means of colorimetric determination in the biological antioxidant potential (BAP) test (Diacron International, Grosseto, Italy). This test enables the measurement of many antioxidants such as uric acid, ascorbic acid, proteins, α-tocopherol, and bilirubin (Benzie and Strain, 1996). The principle of this test involves measuring the BAP of the plasma sample to reduce ferric (Fe^3+^) to ferrous (Fe^2+^) iron. A 10 μL plasma sample is added to a solution of ferric chloride and thiocyanate derivative, and the intensity of any resulting decolorization reflects the ability of the plasma sample to reduce ferric ions [23]. The results are expressed in μmol/L of reduced iron. The degree of oxidative stress was expressed as an oxidative stress index (OSI), where OSI = ROMs/BAP × 100, as this index provides a better indication of redox balance when these measurements are so combined [24,25].

### 2.3. Statistical Analysis

All data (ROMs, BAP, OSI, AOPP, BCS, LW, and milk yield) were analyzed with a REML linear mixed model in GenStat v12 (VSN International Ltd, Hertfordshire, UK) using the following model:

Y_ij_ = μ + α_i_ + B_j_ + e_ijk_,

where Y_ij_ = observation, μ = overall mean, α_i_ = effect of treatment (i = control, YM 250 g, or YM 500 g), B_j_ = effect of cow, and e_ijk_ = random error.

The analysis included between-subjects main effect of treatment, within-subjects main effect of sampling time, and the interaction between time of sampling and treatment; when the effect of the interaction was not significant, it was removed from the model. Cow was included in the model as a random effect, while the others were considered fixed effects. Significant differences were declared at *p* < 0.05. 

## 3. Results

### 3.1. Redox Balance

Plasma concentrations of ROMs, BAP, and AOPP were not influenced by YM supplementation; however, a significant effect of time (*p* < 0.001) was observed. An increase in both ROMs and AOPP concentration was observed over time (Table 1). Plasma ROMs concentration increased slightly after calving and then rose by 15% during Months 1 to 3 after calving. Plasma AOPP concentration was similar in Months −1 and 0, their levels doubled in Months 1 and 4 after calving, while the highest values were observed in Month 3 after calving. Plasma BAP concentrations were relatively similar across the study; however, the lowest values were observed three months after calving. A significant effect of the interaction time of sampling × treatment (*p* < 0.05) was noted on OSI, with both Yerba Mate supplemented groups presenting significantly lower levels that the Control one and three months after calving (Table 1). 

### 3.2. Body Condition Score and Liveweight

No effect of treatment was noted on BCS; however, a significant effect of time (*p* < 0.001) was noted. Following parturition, BCS declined for all groups and was lowest three months after calving, from which point BCS increased back to values observed at calving (Table 2). Changes in LW were not affected by Yerba Mate supplementation, time, or their interaction. 

### 3.3. Milk Yield

**Figure 1:**
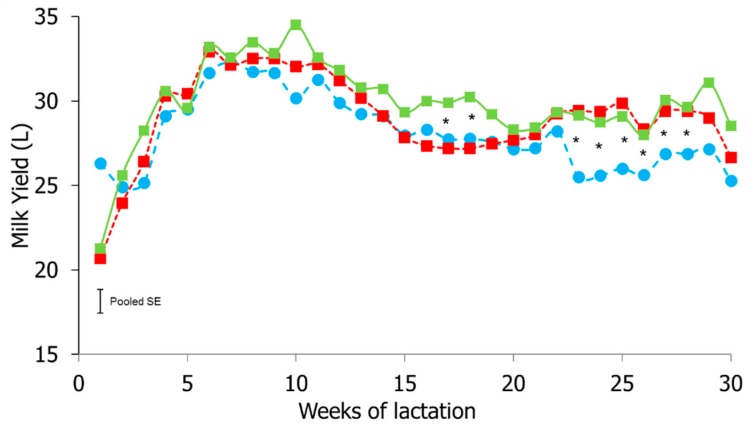
The effect of Yerba Mate supplementation on milk yield in dairy cows. Blue line/circle (Control group), red line/square (Yerba Mate 250), green line/square (Yerba Mate 500). * *p* < 0.05.

Overall, daily milk yield averaged 28.1 ± 0.40, 29.0 ± 0.48, and 29.9 ± 0.46 L/cow/day in the Control, YM 250, and YM 500 groups, respectively. While no significant effect of treatment was observed on milk yield, as expected, milk yield was significantly different over time (*p* < 0.001). Although the YM 500 supplemented cows produced an extra average of 1.79 L of milk per week than the cows in the Control group, this difference was not significant. A significant effect of the interaction time of sampling × treatment (*p* < 0.05) was noted on milk yield, in that the YM 500 group presented higher weekly milk yield levels that the YM 250 and Control groups in Weeks 17 and 18, and that both YM 250 and YM 500 groups had higher weekly milk yield values than the Control group in Weeks 23 to 28 (Figure 1). 

## 4. Discussion

This study suggests that YM supplementation during the dry period seems to have a positive effect on redox balance in dairy cows, as reflected by the lower OSI values observed in the YM fed cows during early lactation. However, in this study, we were not able to observe a direct effect of YM supplementation on either ROMs, BAP, or AOPP concentrations. These findings are in agreement with previous observations where dietary YM supplementation resulted in no changes in ROMs and BAP in mid-lactating cows [16], or in AOPP in growing calves [17]. Therefore, the overall effect of YM supplementation on redox balance seems to be moderate. In dairy ruminants, redox balance biomarkers can be influenced by nutrition and energy balance [26,27,28]. For example, redox balance is disrupted in dairy cows when they are fed high levels of starch [29] and when cows experience excessive energy mobilization [2,30]. The results of this study suggest that there might be limitations when using redox balance biomarkers to assess redox status in vivo. It is clear then that each biomarker of redox balance has its advantages and disadvantages, which is the reason no single measurement can adequately describe redox status. Our findings provide further support for the development of a panel of biomarkers, and combining them in an index that can provide a better representation of redox balance. Therefore, the use of the OSI offers a better indication of redox balance that single biomarkers [24,25].

The observed ranges of AOPP measured in this study are consistent with the values reported in dairy cattle [31,32,33]. AOPP are novel markers of protein oxidation and are predominantly derived from serum albumin and its aggregates damaged by oxidative stress insult [28]. While we were not able to observe an effect of YM supplementation on AOPP concentration, it was significantly affected by time. AOPP are proteins damaged as a result of oxidative stress and arise from the reaction between plasma proteins and chlorinated oxidants mediated by a neutrophil enzyme myeloperoxidase [34,35]. AOPP induce proinflammatory activities and cytokines and increase the circulation of humans in some pathological conditions. AOPP appear to be a by-product of neutrophil activation [36] and are a good indicator of acute inflammation [28]. In agreement with this study, an increase in AOPP concentration has also been observed in dairy cows when they are fed maize silage [16]. Silage is known for its low antioxidant content [37] and, therefore, when high amounts of silage are supplemented in the diet, this could compromise redox balance. Moreover, as rumen bacteria are known to have a reduced antioxidant capacity compared with that of aerobe bacteria, the lower antioxidant content of the silage might also disrupt redox balance in ruminal microbes, reducing the proportion of nutrient channeled towards microbial protein synthesis [38], possibly reducing ruminant production. Indeed, a negative correlation between AOPP concentration and milk yield has been observed in dairy cows [16]. Finally, it is important to note that the observed increase in AOPP during the months when cows received maize silage may indicate the presence of a pro-inflammation event, which could potentially compromise embryonic development [31,32]. Interestingly, the pregnancy rate in the YM supplemented cows was 25%, 50%, and 57% in the control, YM 250, and YM 500 groups, respectively (data not shown), suggesting that YM might have supported embryonic development in dairy cows. However, this finding need to be confirmed in a specifically designed study in which an adequate number of cows is recruited for this purpose [39]. Future studies should also investigate the mode of action by which YM might favor embryonic development because, while the findings of this study indicate that such an effect was not mediated by the modulation of redox balance at the systemic level (blood), it could be possible that changes occur at the local level (uterus and oviducts) [40].

Neither LW nor BCS were affected by YM supplementation; however, a significant effect of time was noted. A loss of LW and BCS was expected in the controls following calving, as this is a normal physiological response to calving and the beginning of lactation. It was surprising to observe that milk yield was higher in the YM fed groups during the last few weeks of this study; however, this finding is in agreement with our previous study, in which YM supplementation during mid lactation sustained milk production [16]. However, other studies were not able to observe any changes in milk yield or composition in dairy cows [18] or sheep [15]. It could be argued that differences in the duration, timing, and dose of the supplementation regime could be responsible for this apparent discrepancy. The effects of YM supplementation on milk yield might be associated with the ruminal effects of the antioxidants contained in YM. Feeding antioxidants to continuous culture fermenters resulted in increased fiber digestibility and microbial N efficiency [41]. In addition, antioxidant supplementation, in the form of ethoxyquin, exerted beneficial effects upon production resulting in increased milk production and organic matter digestibility [42]. Rumen microbes are predominantly anaerobes, which possess a less developed antioxidant capacity than aerobe organisms [43]. Hence, it could be argued that the reduced antioxidant content of silage might have induced oxidative stress in the ruminal microorganisms, compromising their proteolytic activity and optimum growth. Yerba Mate has substantial amounts of potent antioxidants such as polyphenols and caffeoyl derivatives, and its supplementation might have prevented this from occurring. Indeed, in a previous study, we observed that when the silage content of the diet was increased, BAP levels decreased, while ROMs and AOPP increased [16]; furthermore, we also observed that milk yield was positively correlated with BAP level [16], supporting this hypothesis. Future studies are needed to simultaneously evaluate the effects of YM supplementation on rumen fermentation and milk yield.

Hartemink et al. [44] reported that supplementation of YM to dairy cows can influence rumen fiber and protein degradation. The addition of YM in ruminant diet could decrease ammonia production and increase protein availability for production purposes. Therefore, it could be argued that the observed effect of YM on nutrients degradation kinetics might be because of the moderate presence of tannins. It has been reported that YM is rich in hydrolysable tannins [13,45], while it has very little condensed tannins [14]. It has been reported that several production traits such as wool growth, weight gain, milk yield, and ovulation rate can be improved when moderate levels of tannins are included in ruminants’ diet [46]. Considering that the cows in this experiment were fed identical amounts of silage and concentrates, and that pasture availability was restricted, as tannins are known to decrease rumen degradable protein, it is likely that higher levels of dietary protein might have bypassed the rumen. The quantity of dietary and microbial protein flowing from the rumen is a major factor determining the productivity of ruminants. Considering that metabolic adaptations to lactation are initiated late in pregnancy, especially prior to calving [47], it could be argued that YM supplementation may have improved metabolizable protein availability, which may have supported mammary gland development and function. The metabolic adaptations to lactation could help explain subtle increases in milk production that were observed as lactation progressed; however, this will need to be tested in a specific designed study.

Finally, YM also contains caffeine. Caffeine has been demonstrated to increase mammary gland development and milk yield [48]. The increase in mammary gland parenchymal tissue after caffeine administration might have been the result of an increase in cell size and a relative increase in cell numbers, suggesting an increased secretory capacity of the mammary gland [48]. Further work is required to characterize how YM supplementation influences mammary gland function. These studies can lead to the development of novel feeding strategies that exploit the potential beneficial effects of YM. 

## 5. Conclusions

This study suggests that the effect of pre-calving YM supplementation of dairy cows’ diet on redox balance and milk yield appears to be mild. Nutritional manipulation during the dry period with alternative feedstuffs such as Yerba Mate might have the potential to improve production, reproduction, and health; however, this needs to be tested in specifically designed studies.

## Figures and Tables

**Table 1:** The effect of Yerba Mate (YM) supplementation on redox balance in dairy cows.

Item	Diet	Months from Calving					SE	*p*-Value		
		−1	0	1	3	4		Diet	Time	DxT
**ROMs**	Control	105.1	116.6	133.2	130.1	121.4	3.7		***	
	YM 250	104.2	119.6	122.1	121.9	126.6	2.9			
	YM 500	111.1	108.6	110.3	112.6	112.1	2.5			
**BAP**	Control	2560.9	2643.3	2254.2	1954.8	2556.4	95.6		***	
	YM 250	2671.4	3197.4	3152.4	2543.9	2883.4	95.9			
	YM 500	2775.1	2958.3	2600.4	2485.9	3037.5	77.5			
**OSI**	Control	4.96	4.41	5.91 ^a^	6.65 ^a^	4.75	0.4		***	*
	YM 250	3.73	3.74	3.87 ^b^	4.79 ^b^	4.39	0.1			
	YM 500	4.01	3.67	4.23 ^b^	4.53 ^b^	3.69	0.1			
**AOPP**	Control	26.4	16.3	46.4	52.9	51.9	5.5		***	
	YM 250	27.3	24.9	45.5	57.9	48.8	4.8			
	YM 500	26.4	24.4	42.8	61.9	46.6	5.2			

* *p* < 0.05; *** *p* < 0.001. For parameters where a significant effect of the interaction between time of sampling and treatment (Diet x Time) was noted (OSI), means with different superscript letters (^a,b^) indicate significant differences between groups (*p* < 0.05). ROMs—reactive oxygen metabolites; BAP—biological antioxidant potential; OSI—oxidative stress index; AOPP—advanced oxidation protein product.

**Table 2:** Body condition score (BCS) and liveweight (LW) in dairy cows supplemented with Yerba Mate.

Item	Diet	Months from Calving							SE	*p*-Value		
		−1	0	1	3	4	5	6		Diet	Time	DxT
**BCS**	Control	3.4	3.2	2.9	2.7	2.5	2.7	3.1	0.11		***	
	YM 250	3.4	3.3	2.8	2.8	2.7	2.7	2.9	0.10			
	YM 500	3.1	3.3	3.0	2.7	2.6	2.8	3.2	0.09			
**LW**	Control	687	693	624	575	600	602	595	16		***	
	YM 250	655	677	616	589	581	569	576	14			
	YM 500	712	690	655	602	586	600	617	16			

*** *p* < 0.001.
